# Rare involvement of paranasal sinuses in sarcoidosis: case report and literature review

**DOI:** 10.11604/pamj.2019.33.250.16922

**Published:** 2019-07-24

**Authors:** Afnan Rajeh, Andreas Albers, Annett Pudszuhn, Veit Maria Hofmann

**Affiliations:** 1 Department of Otorhinolaryngology, Head and Neck Surgery, Charité-Universitätsmedizin Berlin, Corporate Member of Freie Universität Berlin, Humboldt-Universität zu Berlin, and Berlin Institute of Health, Campus Benjamin Franklin, Berlin, Germany

**Keywords:** Sarcoidosis, paranasal sarcoidosis, sinonasal involvement, treatment of sarcoidosis

## Abstract

Sarcoidosis is a non-caseating inflammatory chronic systemic disease of unknown etiology, which may affect one or more organs. Paranasal sinuses involvement occurs sporadic in sarcoidosis. We report a patient with a medical history of sarcoidosis involving her lungs, liver, and lymphatic system for four years who now presented with nasal and sinuses symptoms. The primary treatment with local cortisone showed no improvement. Computed tomography (CT) scan of the paranasal sinuses (PNS) revealed signs of chronic pansinusitis. She was successfully treated with endoscopic sinonasal surgery. Subsequent histological analysis confirmed the involvement of the PNS with sarcoidosis. Her follow-up during the last 6 months was without recurrence.

## Introduction

Sarcoidosis is a multisystemic inflammatory disease of unknown etiology, which may involve any organ in the body [1-3]. Involvement of lungs and intrathoracic lymph nodes is present in more than 90% of the cases [1,4], while a sinonasal association occurs in less than 5% of the reported sarcoidosis cases [5-10]. Published studies differ in determining the diagnosis of paranasal sarcoidosis depending on symptoms, nasal endoscopy, computed tomography (CT) of sinuses or histology. This report aims to emphasize that even though paranasal sinuses (PNS) involvement is rare, it is an important site of manifestation and symptoms can be treated by functional endoscopic sinus surgery and medication as required by the stage of the disease and nasal or prenasal thickness (PT) involvement respectively.

## Patient and observation

**Figure 1:**
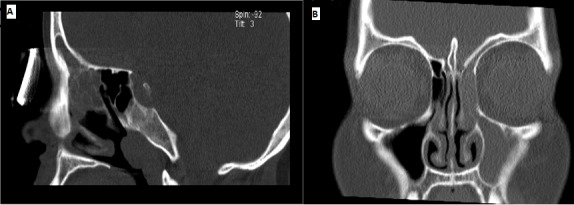
A) sinonasal CT coronal and B) sagittal views in a patient with SNS shows mucosal thickenings of the left maxillary, anterior ethmoidal, and the frontal sinuses as indicated by arrows.

A 31 year old woman presented with left-sided nasal obstruction and pressure pain in the left maxillary sinus and forehead for six months. She was diagnosed four years ago with sarcoidosis with involvement of the lungs and liver, hilar lymphadenopathy, as well as iritis. CT of the PNS showed typical signs of chronic left maxillary, anterior ethmoidal, and frontal sinusitis (Figure 1). Since conservative topical therapy with cortisone had failed, the patient was treated surgically with functional endoscopic sinus surgery (FESS). Histology revealed mucosal granulation with sarcoidosis involvement. The endoscopic follow-up six months after the operation showed no recurrence. After the initial healing phase, the patient reported significant improvement of all symptoms.

## Discussion

While sarcoidosis mainly involves the lower respiratory tract, it rarely affects the PNS. Until 2013 more than 15 studies including about 5000 sarcoidosis patients described occurrence of sinonasal sarcoidosis (SNS) in about 4.5% [1-15]. However, the reported incidence may remain inaccurate for several reasons: either because of the similarity of symptoms compared to nonsarcoid rhinosinusitis, or the physicians lack awareness of such rare involvement by sarcoidosis and therefore inadequate association of sinusitis symptoms with the disease and radiologic diagnostics [7,16]. Bock first reported the SNS in 1905, and the first sinonasal involvement was reported by Schaumann in 1924 and 1926 [17]. A review by Long *et al.* in 2001 of 6 patients with pulmonary sarcoidosis showed four patients with histologically proven SNS [11]. Others found a lower incidence of SNS of 1% out of 1388 of the reported cases, where the significant symptoms were similar to the chronic rhinosinusitis symptoms such as nasal stiffness, rhinorrhea, epistaxis, facial pain, and anosmia [13,15,16].

Histologically, paranasal sarcoidosis is characterized by non-caseating granulomatous inflammation which may occur before systemic manifestation of sarcoidosis [10,15,16]. Reported CT scan findings of the PNS were mucosal thickening, sinus opacification, inflammatories like distraction and bone erosions [2,3,11,14]. In 2004 Braun *et al.* proposed clinical and radiologic criteria for the diagnosis of histologically proven SNS deduced from the study of 15 SNS patients with retrospectively involvement. The radiological criteria were presence of nodules on the septal or turbinate mucosa, nasopharyngeal or pharyngolaryngeal lesions, and complete or subtotal opacification of the sinuses and/or the nasal cavities [3]. Initial treatment of SNS may include topical or systemic steroid therapy and in cases of acute or chronic sinusitis FESS is recommended. The role of FESS in chronic sinonasal sarcoidosis was reported by Kay *et al.* in 2001, where only 7% of 86 patients were treated with FESS [18]. Lately, in 2013, Kirsten A *et al.* presented a retrospective analysis of 12 biopsy-proven cases, where the conservative treatment with topical and systemic corticosteroid following sinuses surgery was successful in most patients. Only 4 of them had repeated surgery because of symptoms persistence [15].

## Conclusion

SNS was found in 4.5% of reported cases worldwide. The clinical presentation included nasal obstruction, epistaxis, rhinorrhea or facial pain. The treatment consisted of local and systemic steroid therapy and in some cases sinus surgery. Sinonasal involvement in patients with sarcoidosis is an important manifestation that can be treated successfully. Thus it should be considered in every cased since SNS may be underdiagnosed.

## Competing interests

The authors declare no competing interests.
